# Viral etiology of pneumonia among severely malnourished under-five children in an urban hospital, Bangladesh

**DOI:** 10.1371/journal.pone.0228329

**Published:** 2020-02-04

**Authors:** Fahmida Chowdhury, Abu Sadat Mohammad Sayeem Bin Shahid, Probir Kumar Ghosh, Mustafizur Rahman, Md. Zakiul Hassan, Zubair Akhtar, S. Mah-E- Muneer, Lubaba Shahrin, Tahmeed Ahmed, Mohammod Jobayer Chisti

**Affiliations:** 1 Infectious Diseases Division, International Centre for Diarrhoeal Disease Research, Bangladesh (icddr,b), Dhaka, Bangladesh; 2 Nutrition and Clinical Services Division, International Centre for Diarrhoeal Disease Research, Bangladesh (icddr,b), Dhaka, Bangladesh; The University of Hong Kong, CHINA

## Abstract

**Background:**

In Bangladesh, pneumonia has a higher mortality among malnourished children aged <5 years. Evaluating pneumonia etiology among malnourished children may help improve empiric treatment guidelines.

**Methods:**

During April 2015—December 2017, we conducted a case-control study among severe acute malnourished (SAM) children aged <5 years admitted to the Dhaka hospital of International Centre for Diarrhoeal Disease Research, Bangladesh (icddr,b). We enrolled hospital admitted SAM children with clinical or radiological pneumonia as cases (during April 2015 to March 2017) and hospital admitted SAM children without any respiratory symptom in the past 10 days before admission as controls (during February 2016 to December 2017). We tested nasopharyngeal wash from both case and control for respiratory syncytial virus (RSV), human metapneumovirus (HMPV), influenza viruses, human parainfluenza viruses (HPIV), rhinovirus and adenovirus by singleplex real-time reverse transcriptase polymerase chain reaction. To identify the independent association of pneumonia with viral pathogens during February 2016 to March 2017, we used multivariable logistic regression for calculating adjusted odds ratios.

**Results:**

We enrolled 360 cases and 334 controls. For case and control the median age was 8 months (IQR: 5–13) and 11 months (IQR: 6–18) (p = 0.001) respectively. Weight/age Z-score was -4.3 (SD ±0.7) for cases and -4.1 (SD ±1.1) for controls (p = 0.01). Among cases 68% had both clinical and radiological pneumonia, 1% had clinical pneumonia and 31% had only radiological pneumonia. Respiratory virus detection was high in cases compared to controls [69.9% (251) vs. 44.8% (148), p = 0.0001]. The most frequently detected viruses among cases were rhinoviruses (79, 22.0%) followed by RSV (32, 8.9%), adenovirus (23, 6.4%), HPIV (22, 6.1%), influenza virus (16, 4.5%), and HMPV (16, 4.5%). Among the controls, rhinoviruses (82, 24.8%) were most commonly detected one followed by adenovirus (26,7.9%), HMPV (5, 1.5%), HPIV (4, 1.2%), RSV (3, 0.9%), and influenza virus (2, 0.6%). RSV (OR 13.1; 95% CI: 1.6, 106.1), influenza virus (OR 8.7; 95% CI: 1.0, 78.9), HPIV (3.8; 95% CI: 1.0, 14.8), and HMPV (2.7; 95% CI: 1.3, 5.5) were independently associated with pneumonia while compared between 178 cases and 174 controls.

**Conclusion:**

Viral etiology of pneumonia in SAM children were mainly attributable to RSV, influenza, HPIV and HMPV. Our study findings may help in planning further studies targeting vaccines or drugs against common respiratory viruses responsible for pneumonia among SAM children.

## Introduction

Pneumonia is the leading cause of morbidity and mortality among children aged less than five years in low and middle income countries [1] where it is commonly associated with malnutrition [2]. In 2016, one in every six childhood deaths was attributed to pneumonia [1]. In 2010, there were an estimated 120 million episodes of pneumonia in children aged less than five years of which 14 million developed severe complications requiring hospitalization [3]. Pneumonia is a common co-morbidity in children presenting with malnutrition and can increase the risk of death 15 fold [2]. Malnourished children might have a blunted inflammatory response, leading to an inability to produce clinical sign-symptoms, despite having infections like pneumonia [4]. An atypical presentation of pneumonia can delay diagnosis and appropriate care, thus increasing the risk of morbidity and mortality in such populations [5].

In Bangladesh, co-morbidity of pneumonia and malnutrition is prevalent and frequently associated with case fatality [6]. Pneumonia accounts for approximately 15% of 1,19,000 deaths among Bangladeshi children aged less than five years [7]. In Bangladesh, 36% of children aged less than five years are malnourished and 12% of them have severe acute malnutrition (SAM) [8]. Two-thirds of the malnourished children admitted to hospital are diagnosed with pneumonia [9]. It is crucial to target malnourished children with pneumonia with the highest risk of death to have a significant impact on global child mortality through proper pneumonia case management [10]. There are scarcity of data about the etiology of pneumonia in children with SAM [2]. Studies suggest that the etiology of pneumonia in malnourished children might be different from that of well-nourished children and predominantly associated with gram-negative bacteria while considering bacterial etiology [2, 5].

In the failure of antibiotics in treating severe pneumonia, one possibility could be non-bacterial aetiology of pneumonia in under-five children. Respiratory viruses are a major cause of childhood pneumonia, especially in young age. It has been estimated that seventy-seven percent of pneumonias among Bangladeshi children aged <2 years were associated with respiratory viral pathogens [11]. The annual incidence of respiratory virus associated hospitalization is approximately 11 per 1000 children aged <5 years and is predominantly associated with respiratory syncytial virus (RSV) illness [12]. Other commonly identified respiratory viruses include influenza viruses, rhinoviruses, adenoviruses, human para influenza viruses (HPIV), and human metapneumoviruses (HMPV) [12]. Relatively little is known, however, about the etiology of viral pneumonia among children with SAM [6].

Antivirals to treat influenza, and influenza vaccines, and monoclonal antibodies to prevent RSV illness are available but seldom used in Bangladesh [13]. Prevention and control efforts in low-income settings are driven by disease burden. Indeed, there are no clinical guidelines for their use in Bangladesh, in part, because their cost-benefit has yet to be established in low-income settings. New antivirals, vaccines, and monoclonal antibodies are also in the primitive stage of development for treating influenza, RSV, and HPIV-3infection [14, 15]. Information about the relative contribution of respiratory pathogens might help health officials to explore the value of targeting such pharmaceuticals to children with severe pneumonia and SAM and to avoid unnecessary antibiotic use. To fill this knowledge gap, we aimed to determine the viral etiology of pneumonia among young children with SAM at an urban hospital in Bangladesh and to also identify the outcome of pneumonia according to different viral etiology within 30 days of hospital admission.

## Methods

### Study setting and patient enrollment

We conducted a case-control study in which we prospectively screened all severely malnourished children aged <5 years admitted to the Dhaka Hospital of International Centre for Diarrhoeal Disease Research Bangladesh (icddr,b) from April 2015 to December 2017. The Dhaka hospital of icddr,b provides care and treatment for approximately 140,000 patients annually mostly from low-socioeconomic urban or peri-urban communities in Dhaka. Sixty percent of patients are aged <5 years admitted with diarrhea only or with diarrhea and other co-morbidities [16]. According to data from Dhaka hospital of icddr,b during 2017, among 6,035 children <5 years with diarrhoea and other co-morbidities around 23% (1,408) were severely malnourished, 24% (1,453) children had pneumonia and 6% (354) had both SAM and pneumonia.

During April 2015 to March 2017, we enrolled children as cases if they were aged <5 years (0–59 months), severely malnourished (i.e., WHO criteria <-3 z score from the median of weight for height/length, weight for age, or nutritional edema) [17] and met WHO clinical criteria for pneumonia [18]. We also enrolled children as cases if they had SAM, cough, and radiological pneumonia. During February 2016 to December 2017, we enrolled SAM children with no pneumonia on admission as controls if they did not have any additional respiratory symptoms and/or signs of pneumonia as classified by the WHO within the past 10 days prior to admission. The only match that was done for our case and control was severe malnutrition and it was done at recruitment. The information was taken from the parents/caregivers. Children who might have chance of migration to outside Dhaka city within a one-month period from admission were not enrolled in the study as we followed-up with enrolled participants during that period to evaluate the death outcome of children with pneumonia related to different viral etiology. Information on chance of migration was validated from the statement of parents/ caregivers.

### Definitions of pneumonia

Pneumonia: If a child presents with severe malnutrition with any sign of pneumonia (any of the WHO defined signs of pneumonia or severe pneumonia or radiological pneumonia) would be considered as pneumonia [19].

Or

A child with severe malnutrition with cough or respiratory difficulty with the presence of end point consolidation or other infiltrates, or pleural effusion in chest X-ray defined by WHO, as assessed by a qualified radiologist [20].

Clinical characteristics of pneumonia: According to WHO, *a c*hild with a history of cough with respiratory difficulty or age-specific fast breathing or lower chest wall indrawing will be defined as pneumonia [18].

Or

According to WHO, a child with a history of cough and/or respiratory difficulty plus oxygen saturation < 90% or central cyanosis, or severe respiratory distress (grunting, very severe chest in-drawing), or signs of pneumonia with a general danger sign (inability to breastfeed or drink, lethargy or reduced level of consciousness, convulsions), auscultatory findings of decreased or bronchial breath sounds or signs of pleural effusion or empyema will be defined as severe pneumonia [18].

### Clinical approach

Study physicians examined each child for chest auscultation and measured length, weight, respiratory rate, oxygen saturation, and axillary temperature. Children with severe malnutrition having cough or respiratory difficulty without any additional sign of pneumonia were evaluated for radiological pneumonia through chest X-ray. These children were enrolled into the study if they met WHO radiological classification of pneumonia [21]. Children with known congenital heart disease presented with features of pneumonia (cough, fast breathing, chest in-drawing and even cyanosis) secondary to congestive cardiac failure. Therefore, these children were further evaluated through chest x-ray and auscultatory findings to diagnose pneumonia. According to the established clinical guidelines of the Dhaka hospital study children were provided nutritional rehabilitation, and treatment of severe pneumonia and hypoxemia (defined as SpO2<90% on pulse oximetry) [18, 22]. Children with severe malnutrition and pneumonia were treated with parenteral ampicillin and gentamicin as the first line of treatment. For those children who did not respond (no improvement within 48 hours or presented deterioration of clinical sign symptoms of pneumonia within 24 hours of initiation of antibiotics) antibiotics were changed to ceftriaxone and levofloxacin according to Dhaka Hospital protocol [23].

### Data collection

Study physicians additionally gathered demographic characteristics including medical history, clinical examination at enrollment, radiological findings, vaccinations and outcome using a structured, pretested case record form. Monthly household income was recorded as a proxy for socio-economic status. Households with a monthly income of <120 USD were considered of low socioeconomic status. The study physician followed up with children within 30 days of admission to assess the outcome of their pneumonia even after discharge from hospital. After recovery and completion of nutritional rehabilitation phase, patients were discharged. The study physician asked caregivers to make weekly follow-up visits to the hospital after discharge from hospital until completion of 30 days from admission in order to monitor the death outcome according to different viral etiology of pneumonia. Transport cost was provided for each follow-up visit. At each follow up visit the study physician asked the mother/caregiver whether there was any sign or symptoms of specific morbidities since the previous visit. Morbidity information like respiratory (runny nose, cough, fever, breathing difficulty, chest indrawing, auscultatory findings), diarrhoeal or others (eye, ear and skin infection, thrush, passage of worms etc) was recorded. Vital signs including z-scores, oedema were recorded at each visit. Following discharge the mother/caregiver was advised to consult with the study physician over cell phone or return to the hospital at any time if any sign or symptoms of illness develop within the scheduled follow up visit.

### Sample collection and laboratory analysis

We collected nasopharyngeal wash (NPW) samples for respiratory viral testing by real-time reverse transcriptase polymerase chain reaction (rRT-PCR) by a trained study physician from 694 cases and controls. Of these collected samples, 5 were invalid samples that were insufficient for testing (1 in case and 4 in controls). Due to higher sensitivity for virus collection NPW was considered as standard specimen collection method instead of nasopharyngeal swab in clinical setting [24]. For sample collection the distal end of the butterfly catheter (needle and butterfly) extension set was cut off so that about 2-3 inches of tubing were left attached to the hub. For the procedure study physician washed hands, wore gown, mask and gloves. Then drew up 4 mls of sterile bacteriostatic saline (0.9% NaCl) in a 5 ml syringe and attached the syringe with the bulb of the catheter. Child was placed in upright position in the mother’s/caregiver's lap and the mother/caregiver restrained arms and legs of the child. Assistant stabilized the child’s head against the mother’s/caregiver's chest and tilted the subject’s head slightly (70°). Then physician inserted the cannula 1.5–2 cm into the nostril parallel to the palate, flushed the saline into posterior nasopharynx, and immediately aspirated back into the syringe and withdrew the cannula. Physician assessed the subject, especially respiratory rate and effort and auscultated the chest; removed the cannula and put aspirate into 2 ml viral transport media (VTM) up to the maximum graduation mark of the collection tube.

The samples were kept in cool box with ice packs at approximately 4°C before being transported to virology laboratory of icddr,b within one hour of sample collection for testing. In virology laboratory two aliquots were prepared from each sample, one was processed for testing and one stored in -80°C freezer for future analysis if needed. Total nucleic acid was extracted from nasopharyngeal wash and rRT-PCR was performed for detecting respiratory viruses using fluorescent Taqman probes. Following this procedure we detected influenza A and B, and their subtypes as well as other common respiratory viruses including respiratory syncytial virus (RSV), adenovirus, rhinovirus, human parainfluenza viruses (HPIV) types1, 2 and 3 and human metapneumovirus (HMPV) using primers and probes designed by CDC (Centers for Disease Control and Prevention), Atlanta as described previously [25, 26]. Chest X-ray and blood culture were obtained only from the case patients.

### Statistical analysis

Assuming 80% power, an alpha of 5%, and 10% drop out among participants, we calculated that we would need 350 cases and controls to distinguish a respiratory viral pathogen detectable in 35% of severely malnourished children with pneumonia and 25% in severely malnourished children without pneumonia [27]. We entered data using SPSS for Windows version 20.0 (SPSS Inc, Chicago, IL), and analyzed it using STATA version 13. We conducted descriptive analysis to summarize the baseline demographic and clinical characteristics and reported frequencies and proportions for categorical variables. For continuous variables we used mean, median, and inter-quartile range (IQR) when appropriate. To examine the statistical significance between case and control patients, we used Chi-squared tests in baseline characteristics. We conducted stratified analysis by age (infants aged < = 12 months and toddlers aged 13–60 months) and performed univariate analysis in etiological characteristics to examine the statistical significance between case (178 children) and control (174 children) enrolled during February 2016 to March 2017. Parainfluenza types 1, 2, and 3 were merged into one category, and influenza virus types A and B were combined into another category for analysis. The monthly detection of respiratory viral pathogens was generated in a trend graph to describe the seasonality among case patients. To identify the independent association of pneumonia with viral pathogens during February 2016 to March 2017, we used multivariable logistic regression for calculating adjusted odds ratios (OR) and 95% confidence intervals (CI). We adjusted for age, PCV vaccination, duration from illness onset to hospitalization, and use of antibiotics prior to hospitalization. Any association with a p value <0.05 was considered statistically significant.

### Ethical consideration

We obtained written informed consent from a parent or a caregiver of the participating children before enrolling them into the study. The study protocol was reviewed and approved by the institutional review boards (IRB; named as Research Review Committee and Ethical Review Committee) of icddr,b. CDC relied on icddr,b’s IRB review.

## Results

**Fig 1:**
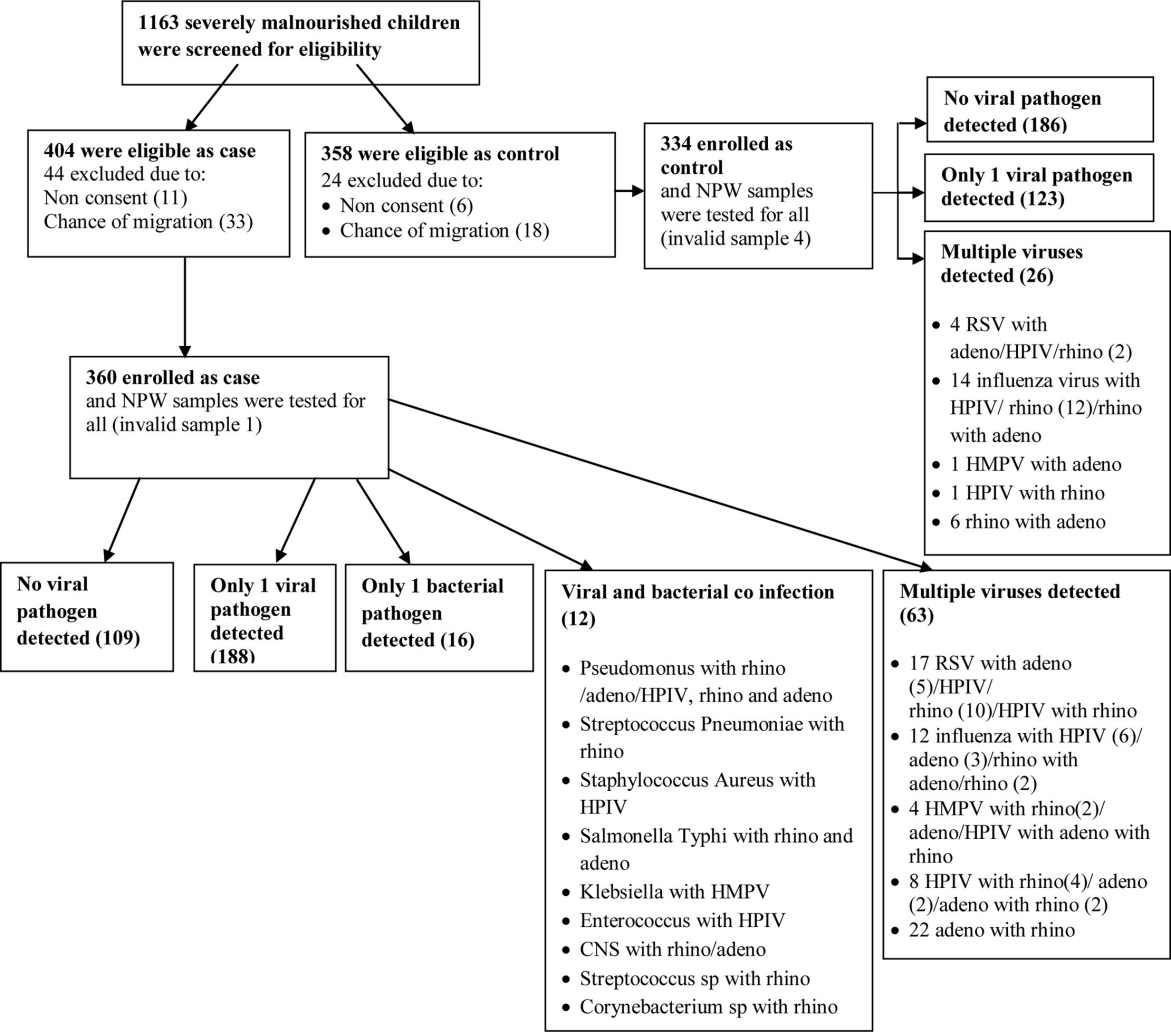
Flowchart on enrolment of study participants and detection of viral and bacterial pathogens. [Abbreviations: NPW-nasopharyngeal wash; RSV- respiratory syncytial virus; HPIV- human parainfluenza virus; HMPV- human metapneumo virus].

We screened a total of 656 SAM children for pneumonia and 507 SAM children with the same criteria for no pneumonia. Of those, 360 children were enrolled as cases and 334 were controls for our study (Fig 1).

Among cases, 68% (245) had both clinical pneumonia and radiological pneumonia, 1% (4) had clinical pneumonia and 31% (111) had only radiological pneumonia. Ninety-nine percent (356) of cases had cough with a median duration of 5 days (IQR: 3–7), 19% (46) had runny nose with a median duration of 3 days (IQR: 2–5), 48% (174) had tachypnoea, 24% (86) had chest indrawing and 13% (45) had hypoxaemia.

For the case and control, median age was 8 months (IQR: 5–13) and 11 months (IQR: 6–18) (p = 0.001) and 62% and 61% were male (p = 0.7), respectively. Younger in age (8 months vs. 11 months, p = 0.001), lower Z score of weight for age (p = 0.01), congenital heart disease (p<0.001), exposure to cigarette smoke at home (p = 0.04), and getting antibiotics before hospitalization (p<0.001) were more common among the cases compared to the controls (Table 1). Pneumococcal Conjugate Vaccine (PCV) vaccination was higher (case: 37% vs. control 61%; p = <0.001)) among controls (Table 1). There was no difference in gender, maternal education and occupation, exclusive breast feeding, weight for height and Bacille Calmette Guerin (BCG) vaccination between cases and controls. A similar proportion of cases and controls had a history of respiratory tract infection (RTI) among family members in last 14 days, household cooked in bedroom, low socioeconomic status, and were slum dwellers (Table 1). Respiratory viruses were detected in 69.9% (251) of 359 cases. The most commonly detected single respiratory virus among cases were rhinovirus 22% (79), followed by RSV 8.9% (32), adenovirus 6.4% (23), HPIV3 5% (18), HMPV 4.5% (16), influenza A virus 3.6% (13), influenza B virus 0.8% (3), HPIV1 0.8% (3), and HPIV2 0.3% (1). Multiple viruses were detected in 17.5% (63) cases (Table 2).

**Table 1:** Socio-demographic and baseline clinical characteristics of severely malnourished under-five children at an urban hospital in Bangladesh.

Baseline Characteristics	Case (N = 360)		Control (N = 334)		*p-value
	n	(%)	n	(%)	
Male	**224**	**62**	**203**	**61**	**0.696**
Age in months, median (IQR)	**8**	**(5–13)**	**11**	**(6–18)**	**0.001**
Maternal education in years					
No formal	**84**	**(23)**	**76**	**(24)**	**0.425**
1–5 years	**135**	**(38)**	**108**	**(32)**	
6–10 years	**108**	**(30)**	**117**	**(35)**	
> = 11 years	**33**	**(9)**	**33**	**(10)**	
Working mother	**43**	**(12)**	**44**	**(13)**	**0.623**
Exclusively breast fed	**75**	**(21)**	**57**	**(17)**	**0.207**
Congenital Heart Disease	**62**	**(17)**	**2**	**(1)**	**<0.001**
Weight for age Z-score (mean ± SD)	**-4.31±0.73**		**-4.13±1.11**		**0.010**
Weight for height Z-score (mean ± SD)	**-2.93±1.40**		**-3.02±1.04**		**0.357**
BCG vaccine	**313**	**(87)**	**298**	**(89)**	**0.853**
Received age appropriate PCV	**134**	**(37)**	**204**	**(61)**	**<0.001**
Received age appropriate pentavalent vaccine	**227**	**(63)**	**238**	**(71)**	**0.085**
History of RTI among family members in last 14 days	**71**	**(20)**	**54**	**(16)**	**0.223**
Cook in bedroom	**6**	**(2)**	**7**	**(2)**	**0.677**
Slum dweller	**43**	**(12)**	**33**	**(10)**	**0.384**
Low socioeconomic status	**228**	**(64)**	**207**	**(63)**	**0.872**
Exposure to cigarette smoke at home	**229**	**(64)**	**187**	**(56)**	**0.041**
First symptom onset on admission in days, median (IQR)	**6**	**(4–8)**	**4**	**(3–5)**	**<0.001**
Received antibiotic before hospitalization	**190**	**(54)**	**98**	**(32)**	**<0.001**
Fever on admission (≥38°C)	**197**	**(55)**	**47**	**(14)**	**<0.001**
Duration of fever on admission (days)	**3**	**(2–5)**	**3**	**(1–4)**	**0.053**
Diarrhoea	**274**	**(76)**	**334**	**(100)**	**<0.001**

*; Chi-square test

**Table 2:** Clinical characteristics of severely malnourished children with pneumonia (case) on admission according to different viral etiology, at an urban hospital in Bangladesh.

Clinical Characteristics	RSV (n = 32) n (%)	Adenovirus (n = 23) n (%)	Influenza virus (n = 16) n (%)	Rhinovirus (n = 79) n (%)	HPIV (n = 22) n (%)	HMPV (n = 16) n (%)	Multiple virus (n = 63) n (%)	No No virus (n = 109) n (%)
Fever	25 (78)	16 (70)	13 (81)	33 (42)	17 (77)	10 (63)	28 (44)	55 (51)
Cough	32 (100)	20 (87)	16 (100)	79 (100)	22 (100)	16 (100)	63 (100)	108 (99)
Runny nose	3 (9)	4 (17)	1 (6)	12 (15)	3 (14)	2 (13)	11 (18)	10 (9)
Tachypnoea	23 (72)	8 (35)	7 (44)	41 (52)	11 (50)	11 (69)	25 (40)	48 (44)
SpO2 <90%	7 (22)	3 (13)	1 (6)	8 (10)	2 (9)	5 (31)	3 (5)	16 (15)
Chest indrawing	11 (34)	6 (26)	3 (19)	18 (23)	6 (27)	4 (25)	12 (19)	26 (24)
Grunting	3 (9)	4 (17)	2 (13)	7 (9)	1 (5)	4 (25)	5 (8)	12 (11)
Head nodding	4 (13)	2 (9)	1 (6)	4 (5)	1 (5)	3 (19)	3 (5)	3 (3)
Temperature (mean, SD)	38.2 (0.67))	38.1 (0.91)	38.4 (0.97)	37.7 (0.90)	38.1 (0.72)	38.1 (1.01)	37.7 (1)	
Rales	26 (81)	7 (30)	9 (56)	46 (58)	15 (68)	13 (81)	33 (52)	52 (48)
Rhonchi	0 (0)	1 (4)	0 (0)	4 (5)	1 (5)	0 (0)	1 (2)	2 (2)
Bronchial breath sound	0 (0)	0 (0)	0 (0)	0 (0)	1 (5)	1 (6)	2 (3)	1 (1)
Wheezing	0 (0)	0 (0)	0 (0)	0 (0)	0 (0)	0 (0)	0 (0)	0 (0)
murmur	6 (19)	5 (22)	1 (6)	17 (22)	3 (14)	3 (19)	7 (11)	20 (19)
**Chest X-ray findings**								
Normal	1 (3)	0 (0)	0 (0)	1 (1)	0 (0)	0 (0)	0 (0)	2 (2)
Infiltrate	29 (91)	23 (100)	16 (100)	74 (94)	21 (96)	14 (88)	60 (95)	102(94)
Consolidation	2 (6)	0 (0)	0 (0)	3 (4)	1 (6)	2 (13)	3 (5)	4 (4)
Other	0 (0)	0 (0)	0 (0)	1 (1)	0 (0)	0 (0)	0 (0)	1 (1)
**Outcome**								
Inpatient death	0 (0)	1 (4)	1 (6)	4 (5)	3 (14)	2 (13)	3 (5)	10 (9)
Post discharge death	0 (0)	1 (4)	1 (6)	2 (3)	1 (5)	0 (0)	3 (5)	4 (4)

Respiratory viruses were detected in 44.8% (148) of 330 controls. The most commonly detected single respiratory virus among controls was also rhinovirus 24.8% (82), followed by adenovirus 7.9% (26), HMPV 1.5% (5), RSV 0.9% (3), HPIV3 0.9% (3), influenza A virus 0.3% (1), HPIV1 0.3% (1), an influenza B virus 0.3% (1). Multiple viruses were detected in 7.9% (26) controls.

Bacterial pathogens were isolated from blood culture in 4% (16) cases. Isolates identified *Pseudomonas* 25% (4), *Enterococcus* 12.5% (2), *Salmonella typhi* 12.5% (2), *Streptococcus pneumoniae* 6.3% (1), *Staphylococcus aureus* 6.3% (1), *Klebsiella* 6.3% (1), *Streptococcus spp* 6.3% (1), *Corynebacterium spp* 6.3% (1),and *Beta-hemolytic streptococcus* 6.3% (1). *Coagulase-negative Staphylococcus* which might be a contaminant was also isolated in 12.5% (2) of children. Antibiotic usage prior to admission was reported in 50% (8) of study children with bacteremia.

**Fig 2:**
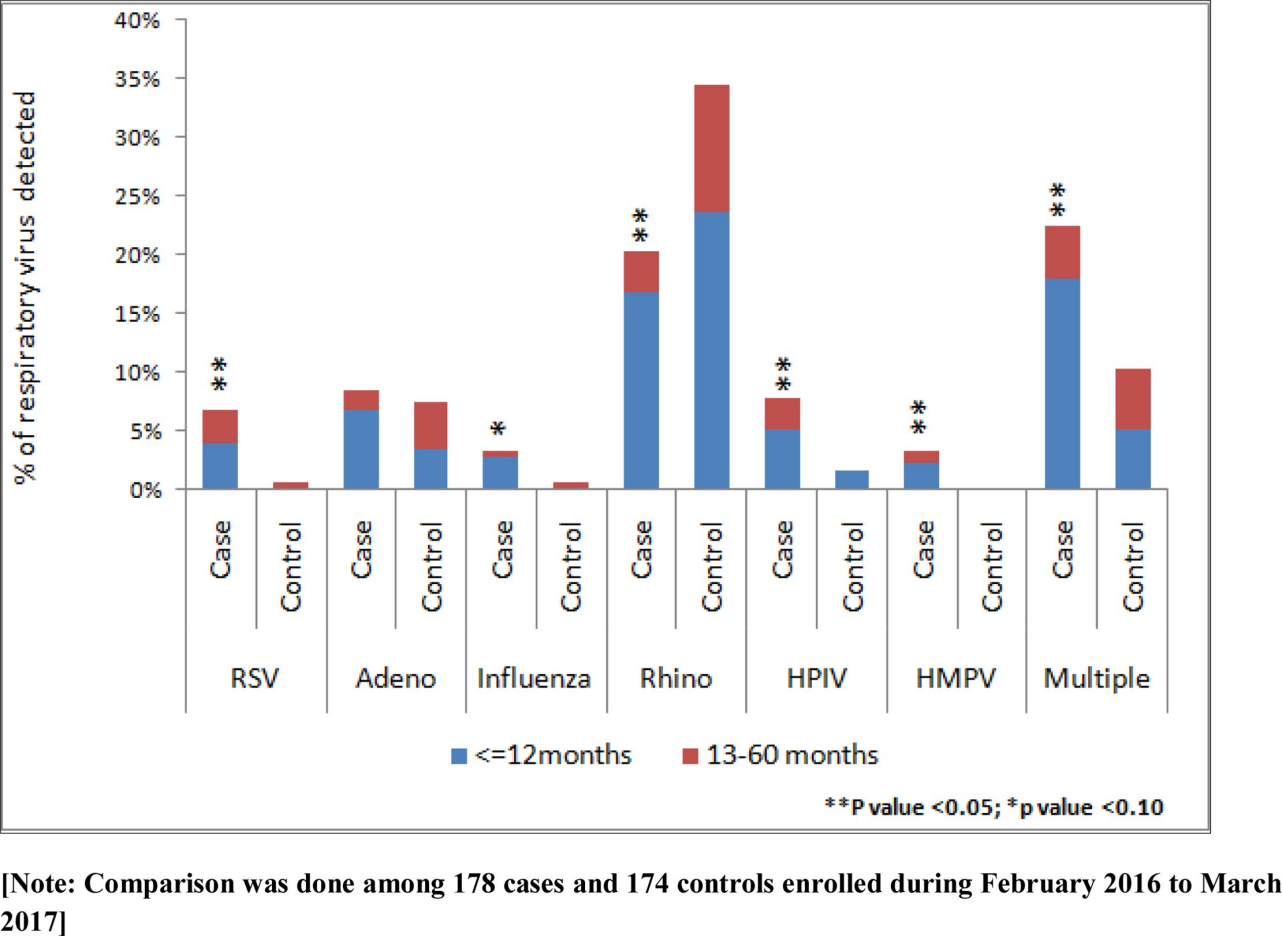
Respiratory viral pathogens detected according to age category among under-five severe acute malnourished children at an urban hospital in Bangladesh [Comparison was done among 178 cases and 174 controls enrolled during February 2016 to March 2017].

During February 2016 to March 2017 we enrolled 178 cases and 174 controls. Detection of respiratory viral pathogens RSV [6.7% (12) vs. 0.6% (1); p = 0.002], influenza virus [3.4% (6) vs. 0.6% (1); p = 0.06], HPIV [7.9% (14) vs. 1.7% (3); p = 0.007], HMPV [3.4% (6) vs. 0% (0), p = 0.02] and multiple viruses [22.5% (40) vs. 10.3% (18); p = 0.002] were higher among the cases when compared to the controls (Fig 2). Detection of adenovirus [8.4% (15) vs. 7.5% (13); p = 0.74) was similar between the groups and rhinovirus [20.2% (36) vs. 34.5% (60); p = 0.003] was higher in controls when compared to cases (Fig 2). Respiratory viral pathogens such as RSV [3.9% (7) vs. 2.9% (5)], adenovirus [6.7% (12) vs. 1.7% (3)], influenza virus [2.8% (5) vs 0.6% (1)], rhinovirus [16.9% (30) vs. 3.5% (6)], HPIV [5.1% (9) vs. 2.9% (5)], HMPV [2.2% (4) vs. 1.1% (2)] and multiple viruses [18.0% (32) vs. 4.6% (8)] were detected more frequently among younger children (< = 12 months vs. 13–60 months) of the cases (Fig 2).

Among the cases, fever was most frequent in children with influenza and subsequently in RSV, HPIV, adenovirus, and HMPV; all the children with respiratory viruses had cough except for those with adenovirus (90%). Compared to other respiratory viruses detected, tachypnea and chest in-drawing were higher in children with RSV; hypoxemia (SpO2<90%), grunting and head nodding were more common in children with HMPV; temperature was higher in children with influenza virus; and rales were more common in children with RSV and HMPV (Table 2). Pulmonary infiltration was reported in all cases of influenza and adenovirus detected. Consolidation was reported mostly in HMPV (Table 2).

Of the enrolled children, 3.7% (26/694; 24 cases and 2 controls) died in the hospital and another 2.0% (14/694; 12 case and 2 control) of the children died during post-discharge follow up period. We had 6.6% (24) inpatient and 3.3% (12) post-discharge deaths among the cases. Among inpatient deaths according to different viral etiology mortality was higher among HPIV (3/22, 13.6%) and HMPV (2/16, 12.5%) detected cases. Then subsequently inpatient deaths were in no virus (10/109, 9.2%), influenza virus (1/16, 6.3%), rhinovirus (4/79, 5.1%), multiple viruses (3/63, 4.8%), adenovirus (1/24, 4.2%) and there was no death in RSV. Post-discharge death was higher in influenza virus detected cases (1/16, 6.3%) followed by HPIV (1/22, 4.5%), multiple viruses (3/63, 4.8%), adenovirus (1/24, 4.2%), no virus (4/109, 3.7%), rhinovirus (2/79, 2.5%) and there was no death in RSV and HMPV detected cases.

**Fig 3:**
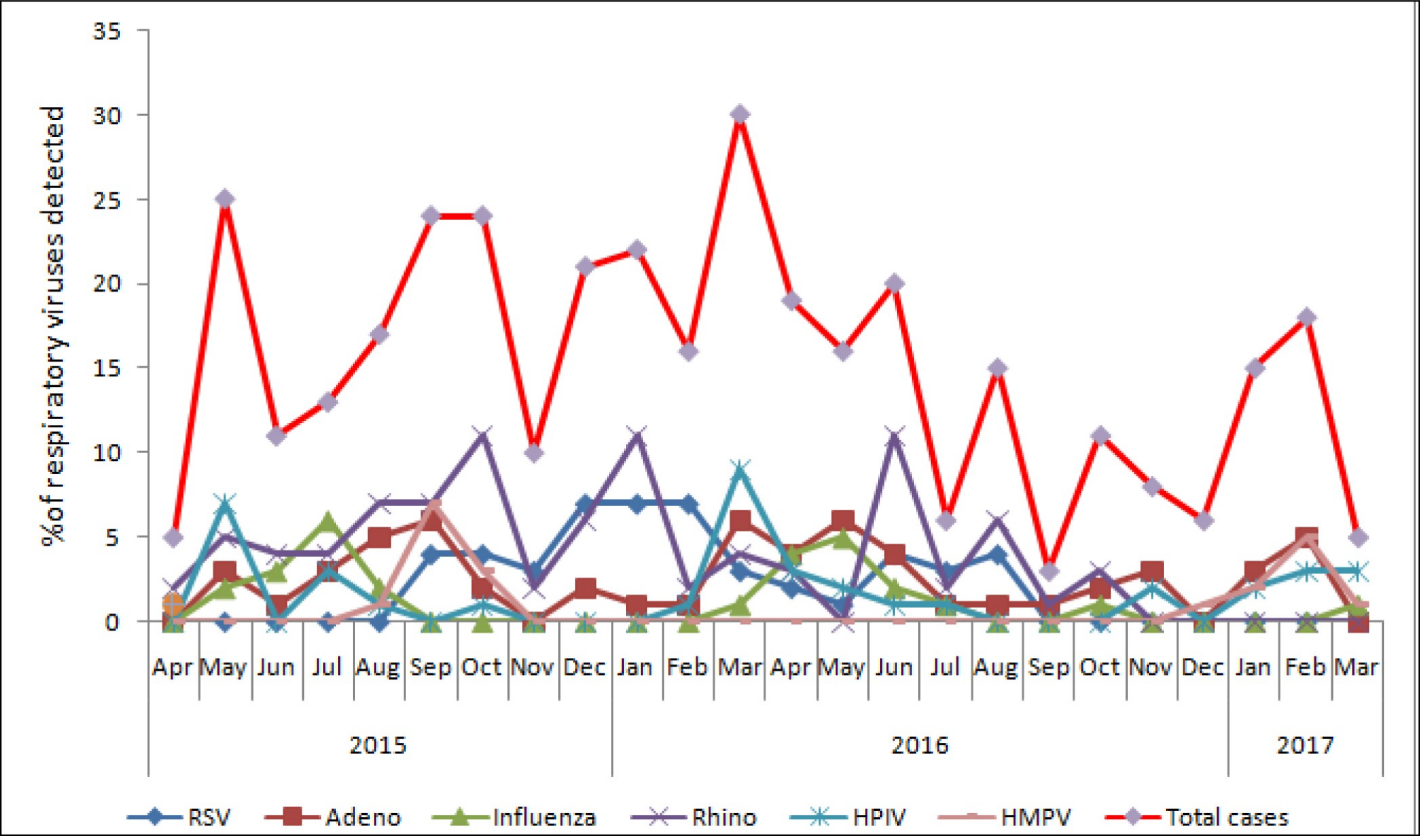
Seasonal detection of respiratory viral pathogens among severely malnourished children with pneumonia (case) between April 2015 to March 2017, at an urban hospital in Bangladesh.

Among the cases, the number of viral pathogens during specific months of the two year study period is demonstrated in Fig 3. RSV was detected constantly from September 2015 to August 2016, with a peak in winter from December 2015 to February 2016. Influenza virus was detected during the already defined influenza season in Bangladesh (May to October). HPIV was detected throughout the year, with a first peak in May 2015 and a second peak in March 2016. HMPV was detected from August to October in 2015 with a peak in September and from December 2016 to March 2017 with a peak in February. Adenoviruses and rhinoviruses were detected throughout the year without any seasonal variation.

After adjusting for age, PCV vaccination, duration of illness onset to hospitalization, and use of antibiotics prior to hospitalization, RSV (OR 13.1; 95% CI: 1.6, 106.1), influenza (OR 8.7; 95% CI: 1.0, 78.9), HPIV (3.8; 95% CI: 1.0, 14.8), and HMPV (2.7; 95% CI: 1.3, 5.5) were identified as the independent viral pathogens for pneumonia in severely malnourished children (Table 3).

**Table 3:** Independently associated respiratory viral pathogens with pneumonia in severely malnourished under-five children, at an urban hospital in Bangladesh.

Respiratory Viral pathogens	Odds Ratio (95% CI)	Adjusted Odds Ratio (95% CI)	P value
Respiratory syncytial virus (RSV)	12.5 (1.6–97.3)	13.1 (1.6–106.1)	0.016
Adenovirus	1.1 (0.5–2.5)	1.4 (0.6–3.5)	0.424
Influenza virus	6.0 (0.7–50.7)	8.7 (1.0–78.9)	0.055
Rhinovirus	0.5 (0.3–0.8)	0.7 (0.4–1.4)	0.332
Human parainfluenza virus (HPIV)	4.9 (1.4–17.2)	3.8 (1.0–14.8)	0.055
Human metapneumovirus (HMPV)	2.5 (1.4–4.6)	2.7 (1.3–5.5)	0.006

Adjusted for age, PCV vaccination, duration of illness onset to hospitalization, and use of antibiotics prior to hospitalization

[Logistic regression analysis was done among 178 cases and 174 controls enrolled during February 2016 to March 2017]

## Discussion

The most important observation of this study was the independent association of RSV, Influenza, HPIV, and HMPV with pneumonia among children with SAM. Our observation was consistent with previous studies that showed the same association in well-nourished children while compared to healthy controls [28–32]. We detected at least one laboratory confirmed respiratory virus in 70% of the study children hospitalized with pneumonia and in 44% of the study children without pneumonia. To our knowledge our study was the first prospective case-control study that described the common respiratory viral pathogens associated with pneumonia in severely malnourished <5 children living in Bangladesh. The only other study on viral etiology of pneumonia in malnourished children was conducted in Gambia in 1994 and detected 35% viral etiology in malnourished children with pneumonia and 25% in children without pneumonia, which is much lower than what we have found in our investigation [27]. A recent study in Ecuadorian children also found an association between being underweight and increased odds of RSV[33]. The detection of influenza virus among our study population is also found to be consistent with previous studies involving children with severe pneumonia [33, 34]. It is important to note that in developed countries respiratory viruses were detected in up to 81% of young children with pneumonia, with RSVbeing the most frequently detected virus in most studies, ranging from 7% to 48% [35–37]. HMPV was more frequently detected in community acquired pneumonia in high-income countries, ranging from 23% in USA to 15% in Sweden [33, 38, 39], and was less frequent in low-and middle-income countries ranging from 3% in Kenya to 10% in South Africa [40, 41], which coincides with our study findings. In developing countries studies have shown 37% to 97% detection of respiratory viruses in children with pneumonia and 7% to 82% in children without any respiratory symptom [28–32]. As our main aim was to evaluate viral etiology of pneumonia in SAM children because of lack of previous data, our study results demonstrate that RSV was the most common viral pathogen causing pneumonia. Although we did not have any control of well nourished children with pneumonia recently published PERCH data from Bangladesh site and other developing countries revealed the predominance of RSV as the main viral pathogen causing pneumonia [42]. The spectrum and frequency of other pathogens detected such as HMPV, HPIV, influenza virus were consistent with PERCH data. The wide range of respiratory viruses detected among asymptomatic control children raises the concern of independent respiratory virus associated pneumonia.

Rhinovirus and adenovirus were the most frequently detected viruses and their rates of detection were similar in both the cases and the controls. Rhinovirus was detected in nearly one-third of all study children indicating a lower likelihood of rhinovirus being a casual pathogen of pneumonia in severely malnourished children. Similarly, in the USA and Alaska respectively, rhinovirus was detected in 27% and 44% of children with pneumonia requiring hospitalization and 17% and 33% in asymptomatic control children [28, 36]. Despite literature supporting the association of human rhinovirus with pneumonia [43], the continued shedding of human rhinovirus even two weeks after infection makes it challenging to infer a causal association between rhinovirus and pneumonia [44]. Adenovirus was detected in almost one-fourth of all study children and thus could not be associated with pneumonia. Similarly, previous studies have shown detection of adenovirus in 21 to 30% of children with pneumonia and in16% of children without any respiratory symptoms [28, 45], despite adenovirus being a potential cause of pneumonia in young children [46]. We found that three-fourths of our study children with pneumonia and all children in the control group without pneumonia had acute diarrhoea. As adenovirus is known to be an important viral cause of acute diarrhoea [47], its detection in the control group might be due to acute diarrhoea or due to co-infections with other viral and bacterial pathogens [33]. However, we did not test stool samples from our study population to explore an association between adenovirus and diarrhoea. The contribution of rhinovirus and adenovirus to independent etiology of pneumonia in malnourished children remains uncertain and needs further investigation.

The presence of respiratory viral pathogens in asymptomatic healthy children in case control studies makes interpreting causative pathogens for pneumonia challenging and obscures its clinical significance [48]. This draws attention to the importance of representative controls as a reference when assessing the impact of respiratory viruses on the development of pneumonia. Both the cases and the controls were from the same low socioeconomic urban and peri-urban communities of Dhaka city and were enrolled after hospital admission according to eligibility criteria. However, it might be interesting to correlate the threshold cycle (Ct) values obtained from RT-PCR to identify more precisely the relationship between a detected viral pathogen and pneumonia etiology [49].

Different respiratory viral pathogens were detected more frequently in cases less than 1 year of age highlighting the importance of vaccination in younger age groups. In Ecuadorian children RSV and HPIV were significantly higher among younger children [33]. Another study in Kenya reported the highest incidence of respiratory virus associated admission for pneumonia among infants [30]. Our study findings of 6.7% inpatient death in SAM children with pneumonia were similar to other developing countries, such as Kenya (5.9%) and India (8.2%) [50, 51]. According to viral etiology, mortality was higher among cases with HPIV (14%) and HMPV (13%). Studies have shown HMPV infection was associated with higher severity of illness in hospitalized children with pneumonia with fatal outcome [52, 53]. Hypoxemia, grunting and head nodding were more common in HMPV infection compared to other respiratory viruses indicating higher disease severity. In contrast, though mortality was higher in HPIV, hypoxemia, grunting, and head nodding on admission in terms of disease severity was not higher in this group compared to other respiratory viral pathogens. Apart from HMPV clinical signs of chest indrawing, however, hopoxemia and head nodding were common in cases with RSV infection compared to other respiratory viruses but no death was observed in this group. RSV was found to be associated with disease severity in children with pneumonia [30].

It was surprising that in this study, RSV was detected simultaneously for only a year within the two-year study period of children hospitalized with pneumonia. However, peak was observed in winter during December to February, which was similar to what some other countries reported in a recent review article on RSV seasonality [54]. The review article reported that, epidemic in different countries were consistent between different years with some changes between seasons observed from year to year. Nevertheless, other countries reported irregular patterns of RSV seasonality as 2 different seasons for RSV and also followed a two-year cycle for RSV. Influenza virus was detected mostly during the influenza epidemic (May to September) in Bangladesh identified through the influenza surveillance system [55]. No definite seasonal pattern was observed for other respiratory viruses.

Despite the inclusion of the PCV and Hib vaccines in the EPI programme in Bangladesh, viral pneumonia continues to be a major contributor of childhood pneumonia. The treatment of viral infections is often challenging due to the empirical use of antibiotics or antivirals. Currently, the development of an RSV vaccine is in progress and is modified for target populations such as infants, children, and pregnant women [56]. In developing countries, RSV causes significant morbidity and mortality, as well as economic burden among children highlighting the crucial need for RSV vaccine implementation [57]. In developed countries, the influenza vaccine is recommended for children, but vaccination coverage remains inadequate [58]. In developing countries with a high burden of childhood pneumonia, such as Bangladesh, the necessity for influenza vaccination among children needs to be considered [59]. For the treatment and prevention of pneumonia among severely malnourished children our study has generated imperative substantiation for physicians and policy-makers to plan and develop enhanced strategies through targeting vaccines and antivirals for common causative respiratory viral pathogens of pneumonia. Further research should be in consideration targeting vaccines or drugs to reduce the pneumonia burden in this specific population.

## Limitations

The main limitation of our study was that we failed to enroll well-nourished controls. As a result we were unable to show the representativeness of the controls. This may reduce the generalizability of the causality from microbiological findings. On the other hand, introduction of hospital-based controls which differs in exposure compared to the cases may weaken the causality from a microbiological point of view. Another limitation of our study was that we did not enroll both the case and control during the same time period and the seasonal circulation of respiratory pathogens may have impacted our findings. However, we have a one year (February 2016 to March 2017) overlapping time period for both case and control enrollment and thereby we conducted analysis during this time period to identify etiological and independent causal associations of pneumonia. Moreover, we did not subtype the predominant rhinoviruses and adenoviruses to identify the pathogenic subtype causing pneumonia. Another limitation of our study was that we did not test for other respiratory viruses such as bocavirus, coronavirus, cytomegalovirus and enterovirus which also might have an impact in our study findings. Another limitation of the study is to perform bacterial culture only for blood which has traditionally low yield and not to perform bacterial culture for all NPW that were used for viral culture and thus, the chance of bacterial co-infection in these children with pneumonia could not be eliminated. Finally, as the study was conducted in a diarrheal disease hospital in Dhaka city, the external validity of our study is limited.

The major strength of our study was the study design, in particular the inclusion of respiratory symptom-free controls, to assess the pneumonia etiology by comparing the detection of pathogens in both groups, as inclusion of control in searching for the viral etiology of pneumonia specifically in severely malnourished children is happening for the first time. Another strength of this study was the well-trained team that evaluated and documented respiratory sign symptoms and used the standardized definition of pneumonia and laboratory measurement techniques.

## Conclusion

Pneumonia in severely malnourished children was mainly attributable to RSV, influenza, HPIV and HMPV. Rhinovirus and adenovirus were detected in over two-thirds of the respiratory symptom-free control children, so identifying their contribution to pneumonia etiology was challenging. This study provides important evidence for clinicians as well as for the public health policy makers to plan and develop improved strategies for the treatment and prevention of pneumonia among severely malnourished children. Our study findings may help in planning further studies targeting vaccines or drugs against common viruses responsible for pneumonia in these children.

## Supporting information

S1 Data File (SAV)
